# Murine models of HRAS-mediated cutaneous skeletal hypophosphatemia syndrome suggest bone as the FGF23 excess source

**DOI:** 10.1172/JCI159330

**Published:** 2023-05-01

**Authors:** Diana Ovejero, Zachary Michel, Christophe Cataisson, Amanda Saikali, Rebeca Galisteo, Stuart H. Yuspa, Michael T. Collins, Luis F. de Castro

**Affiliations:** 1 Musculoskeletal Research Unit, Hospital del Mar Medical Research Institute (IMIM), Barcelona, Spain.; 2 Skeletal Disorders and Mineral Homeostasis Section, National Institute of Dental and Craniofacial Research (NIDCR), NIH, Bethesda, Maryland, USA.; 3 Laboratory of Cancer Biology and Genetics, National Cancer Institute (NCI), NIH, Bethesda, Maryland, USA.

**Keywords:** Bone Biology, Endocrinology, Bone disease, Mouse models

## Abstract

Cutaneous skeletal hypophosphatemia syndrome (CSHS) is a mosaic RASopathy characterized by the association of dysplastic skeletal lesions, congenital skin nevi of epidermal and/or melanocytic origin, and FGF23-mediated hypophosphatemia. The primary physiological source of circulating FGF23 is bone cells. However, several reports have suggested skin lesions as the source of excess FGF23 in CSHS. Consequently, without convincing evidence of efficacy, many patients with CSHS have undergone painful removal of cutaneous lesions in an effort to normalize blood phosphate levels. This study aims to elucidate whether the source of FGF23 excess in CSHS is RAS mutation–bearing bone or skin lesions. Toward this end, we analyzed the expression and activity of *Fgf23* in two mouse models expressing similar *HRAS/Hras* activating mutations in a mosaic-like fashion in either bone or epidermal tissue. We found that HRAS hyperactivity in bone, not skin, caused excess of bioactive intact FGF23, hypophosphatemia, and osteomalacia. Our findings support RAS-mutated dysplastic bone as the primary source of physiologically active FGF23 excess in patients with CSHS. This evidence informs the care of patients with CSHS, arguing against the practice of nevi removal to decrease circulating, physiologically active FGF23.

## Graphical abstract

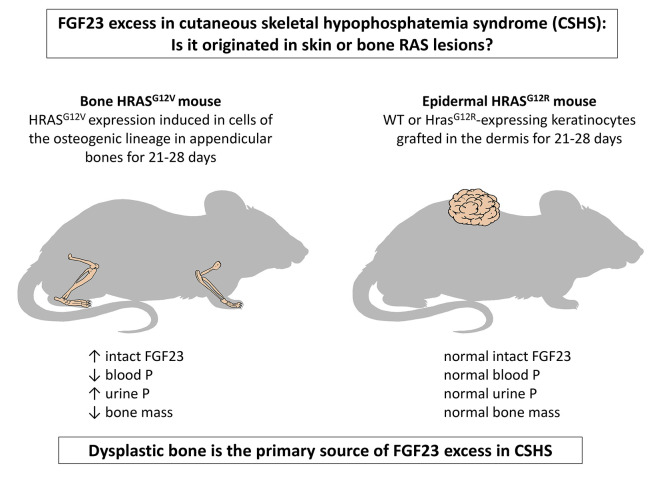

## Introduction

Cutaneous skeletal hypophosphatemia syndrome (CSHS) is characterized by the association of dysplastic skeletal lesions, congenital skin nevi of epidermal and/or melanocytic origin (including epidermal nevi [EN], giant congenital melanocytic nevi [GCMN], and phacomatosis pigmentokeratotica [PPK]), and FGF23-mediated hypophosphatemia (1). It is a mosaic disorder that arises from de novo activating *RAS* mutations during early embryonic development, affecting primarily bone and skin, although involvement of other tissues, including the central nervous system, is frequently identified (2). To date, only gain-of-function mutations in codons 13 and 61 in *HRAS* and *NRAS* genes have been identified in CSHS.

While the clinical spectrum of CSHS varies greatly from individual to individual in terms of the extent and location of bone and skin lesions and the degree of FGF23-mediated hypophosphatemia, in the majority of patients, the most debilitating manifestation is hypophosphatemia (2). FGF23 is a bone-derived hormone that plays a key role in phosphate homeostasis by downregulating sodium-phosphate cotransporters and 25-hydroxyvitamin D3 1α-hydroxylase in proximal renal tubule cells, leading to increased urinary phosphate excretion and decreased production of active 1,25(OH)_2_ vitamin D3, respectively (3). Thus, sustained and uncontrolled FGF23 production results in chronic hypophosphatemia, which ultimately causes muscle weakness, osteomalacia, bone pain, fractures, deformities, rickets, and growth impairment in children (4). Oral phosphate and active vitamin D supplements constitute the first line of treatment for FGF23-mediated disorders, including CSHS (4). However, this drug regimen is often insufficient for attaining adequate management of hypophosphatemia-derived manifestations for many patients with CSHS (2). In addition, this regimen is difficult to adhere to and commonly causes secondary or tertiary hyperparathyroidism and nephrocalcinosis (4). Consequently, alternative treatments have been pursued, including the resection of skin nevi under the assumption they were the source of a phosphaturic factor (FGF23) as well as burosumab, an anti-FGF23 monoclonal antibody drug (5).

In 1977, before the discovery of FGF23, Aschinberg et al. reported the case of a boy with severe hypophosphatemic rickets and skin lesions consistent with PPK, a syndromic association of a variety of cutaneous lesions, in addition to EN. Several angiofibromas, not EN, were resected from the boy, after which a poorly defined improvement of blood phosphate was reported (6). The authors asserted the skin lesions were the source of a “phosphaturic substance” and postulated that surgical removal of skin lesions could treat phosphate disturbances in these patients. Since then, additional publications, some of them relatively recent, presented cases of patients with CSHS who were subject to nevi removal, an often-painful procedure, in an effort to treat hypophosphatemia (2, 7–18). Some reported apparent positive outcomes (7–14, 17, 18), but rigorous review of these cases indicate that most of these patients were also receiving phosphate and vitamin D supplementation at the time of the intervention, confounding interpretation of the impact of removal of skin lesions on improvement in phosphate homeostasis. Given that bone is the source of FGF23 in both physiologic and pathologic conditions such as fibrous dysplasia (FD) (19–21) and that the vast majority of patients with extensive EN or GCMN caused by the same gain-of-function *RAS* mutations do not have hypophosphatemia (22, 23), we postulate that dysplastic bone is the tissue source of the “phosphaturic substance” proposed by Aschinberg et al. and now identified as FGF23.

**Figure 1:**
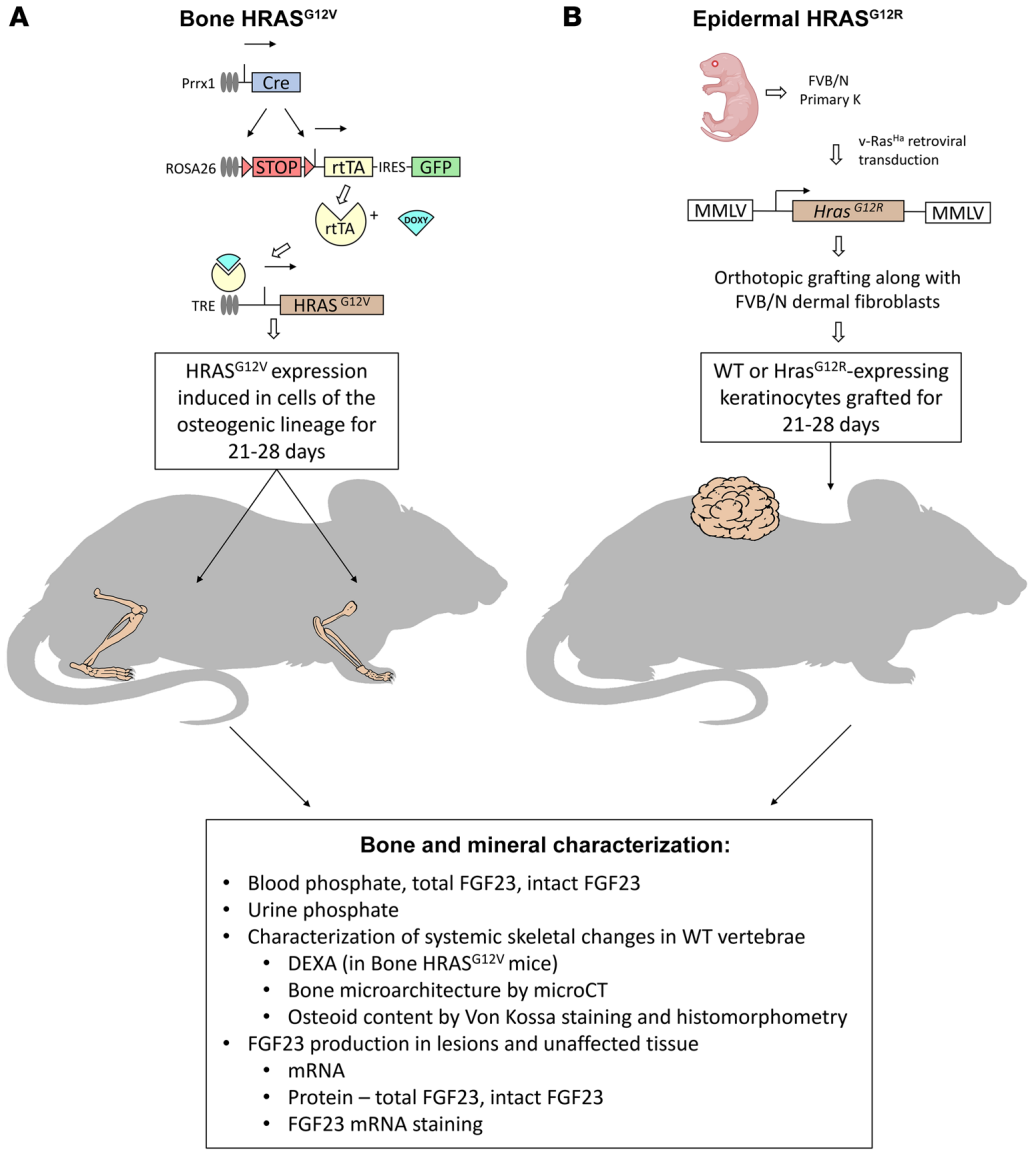
Experimental schematic overview of CSHS mouse models. (**A**) Bone HRAS^G12V^ mouse is a triple transgenic mouse. During embryogenesis, the Prrx1 promoter is activated in the limb budding mesenchyme, driving expression of Cre that excises the LoxP-STOP-LoxP sequence, preventing the constitutive expression of rtTA. GFP is also expressed together with rtTA through an IRES element. When adult mice are fed doxycycline-supplemented food, rtTA becomes active, initiating the expression of HRAS^G12V^ in the cells lacking the LoxP-STOP-LoxP sequence, mainly all the osteogenic lineage cells in the appendicular skeleton. During induction, vertebral bone mass is tracked through DEXA, and after 21 to 28 days of HRAS^G12V^ expression, mice are euthanized and characterized. (**B**) The Epi *Hras^G12R^* mouse is obtained by an orthotopic graft of *Hras^G12R^*-expressing keratinocytes. First, primary mouse keratinocytes are isolated from newborn pups of FVB/NCr, cultured, and transfected with the v-ras^Ha^ retrovirus. Before grafting, transfected keratinocytes are mixed with embryonic primary dermal fibroblasts. At 21 to 28 days after transfection, mice develop skin lesions and are euthanized and characterized. For the epidermal WT control group, littermate mice underwent a similar procedure, but the keratinocytes were not transfected.

Elucidating the source of pathological FGF23 production in CSHS has important clinical consequences; nevi removal is both painful and associated with risk for patients, typically children, especially since evidence that it corrects hypophosphatemia is lacking. To this end, we studied FGF23 production and expression, phosphate homeostasis, and bone density and microarchitecture in 2 mouse models that independently recapitulate the skeletal and epidermal aspects of CSHS (Figure 1). To obtain a mosaic-like expression of *HRAS* mutations in bone, we developed the bone *HRAS*^G12V^ mouse, characterized by inducible expression of *HRAS*^G12V^ in the appendicular skeleton. For the epidermal *Hras^G12R^* (Epi *Hras^G12R^*) mouse, we used an orthotopic grafting assay, in which a papillomatous lesion forms following the transplantation of *Hras^G12R^*-transduced mouse primary keratinocytes on a WT recipient (24).

Our findings strongly support the notion that hypophosphatemia and rickets in CSHS is due to excessive release of FGF23 by mutation-bearing bone lesions. This report aims to settle the debate as to the source of excessive FGF23 in CSHS and free patients from painful, unnecessary, and ineffective treatments.

## Results

### HRAS p.G12V expression in dysplastic skeletal lesions increased circulating FGF23, causing hypophosphatemia and osteomalacia.

**Figure 2:**
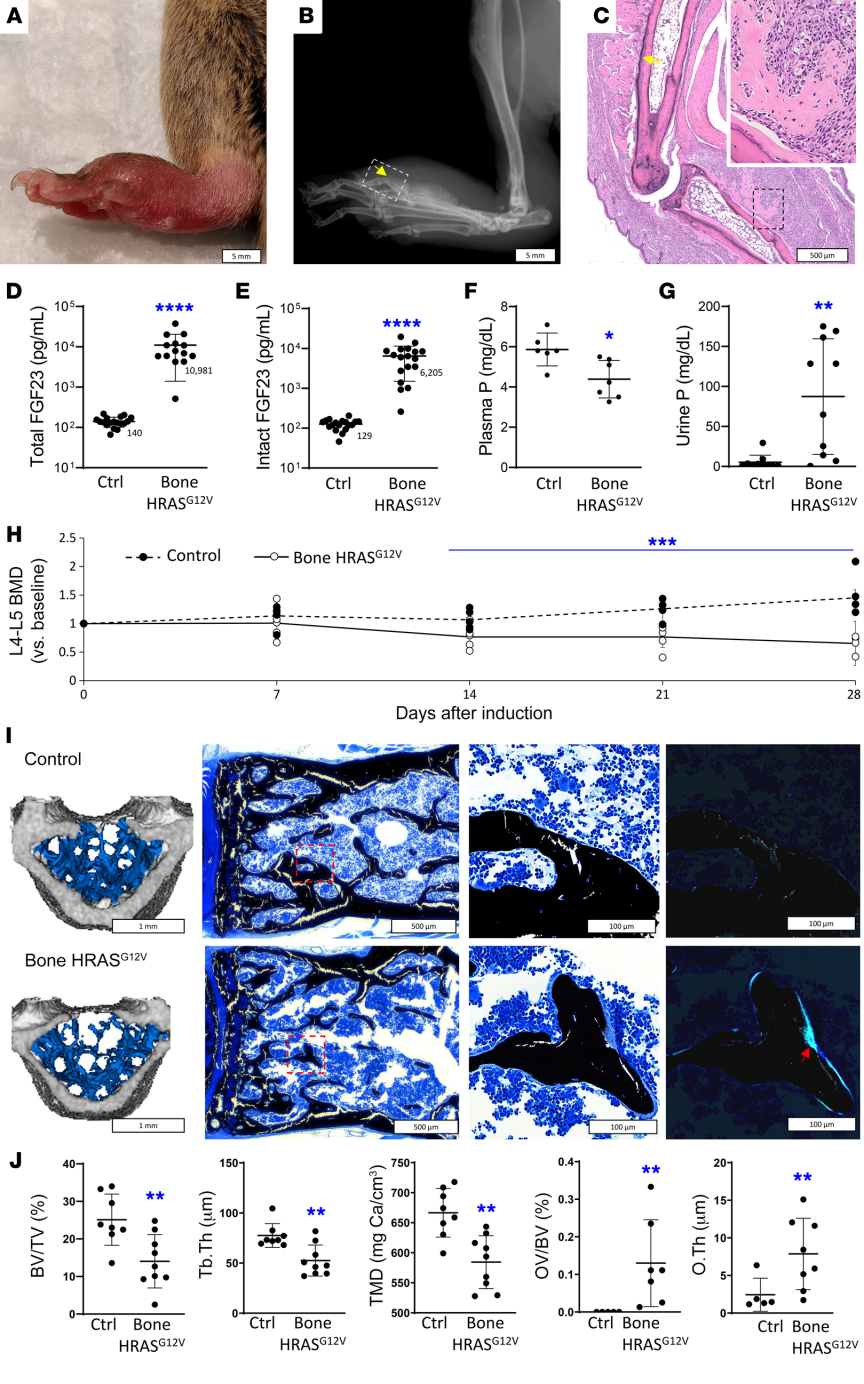
Bone HRAS^G12V^ mice have increased levels of FGF23, leading to hypophosphatemia and osteomalacia. Representative external photography (**A**), radiography (**B**), and H&E histology (**C**) of a paw 28 days after induction of HRAS^G12V^ expression by bone cells with doxycycline-supplemented diet. Swelling shown in **A** is due to the formation of a partially mineralized mass arising from the metatarsals and phalanges periosteum (yellow arrows). White box in **B** represents the histology field in **C**, showing densely packed, highly proliferative fibrotic tissue in the black box magnified in the top right corner. (**D**) Plasmatic levels of total (intact and C-terminal fragments) FGF23 (*n* = 14–18). (**E**) Plasma levels of intact FGF23 (*n* = 18). (**F**) Plasma levels of phosphate (*n* = 6–7). (**G**) Urinary levels of phosphate (P) (*n* = 10). (**H**) Bone mineral density (BMD) in vertebrae L4 and L5, as measured by DEXA and expressed as fold-change per mouse with respect to preinduction baseline (*n* = 5). (**I**) Representative images, left to right: μCT reconstruction of L5 vertebrae, showing trabecular bone loss in bone HRAS^G12V^ mice versus controls (blue region); von Kossa stain at low magnification with a red box showing the areas magnified to the right; far right shows the same areas under polarized light microscopy with a red arrow showing extensive osteoid rims in bone HRAS^G12V^ mice. (**J**) μCT bone microarchitecture parameters. Tb Th, average trabeculae thickness; TMD, tissue mineral density; OV/TV, osteoid volume per total bone volume; O Th, average osteoid rims thickness (*n* = 5–8). Data are represented as individual values and averages or averages with SD bars. *P < 0.05; ***P < 0.001; ****P < 0.0001, Mann-Whitney *U* test (**D** and **E**); 2-tailed Student’s *t* test (**F**–**H** and **J**).

Upon induction with doxycycline, mice rapidly developed distended, fluctuant lesions in the paws (Figure 2A). X-rays demonstrated a poorly mineralized mass arising from the periosteum underlying the swelling that resembled a periosteal osteosarcoma (Figure 2B). Histological examination of these areas demonstrated edema and dysplastic tissue arising from the periosteum, also reminiscent of a periosteal osteosarcoma (Figure 2C) (25).

Bone *HRAS^G12V^* mice had markedly increased blood levels of FGF23 relative to WT, with a 52-fold increase in total FGF23 and 38-fold increase in intact FGF23 (Figure 2, D and E). This was associated with significantly lower blood phosphate and increased urine phosphate levels relative to WT (Figure 2, F and G). Findings were similar when the mice were euthanized at 21 to 28 days after induction.

Starting 2 weeks after induction, bone *HRAS^G12V^* mice progressively lost bone mass in the spine, a skeletal site that does not express *HRAS^G12V^*, consistent with a systemic, not local, effect of hypophosphatemia (Figure 2H). This was further characterized by microcomputed tomography (μCT) analysis of vertebra L5, in which marked decreases in bone volume per total volume (BV/TV), tissue mineral density, and other bone microarchitectural parameters were measured 28 days after induction (Figure 2, I and J). Von Kossa (VK) staining of the vertebrae revealed extensive osteoid rims in the trabeculae, better visualized using polarized light microscopy and indicative of osteomalacia (Figure 2, I and J).

### Epi Hras^G12R^ mice had normal intact FGF23 and blood phosphate levels.

**Figure 3:**
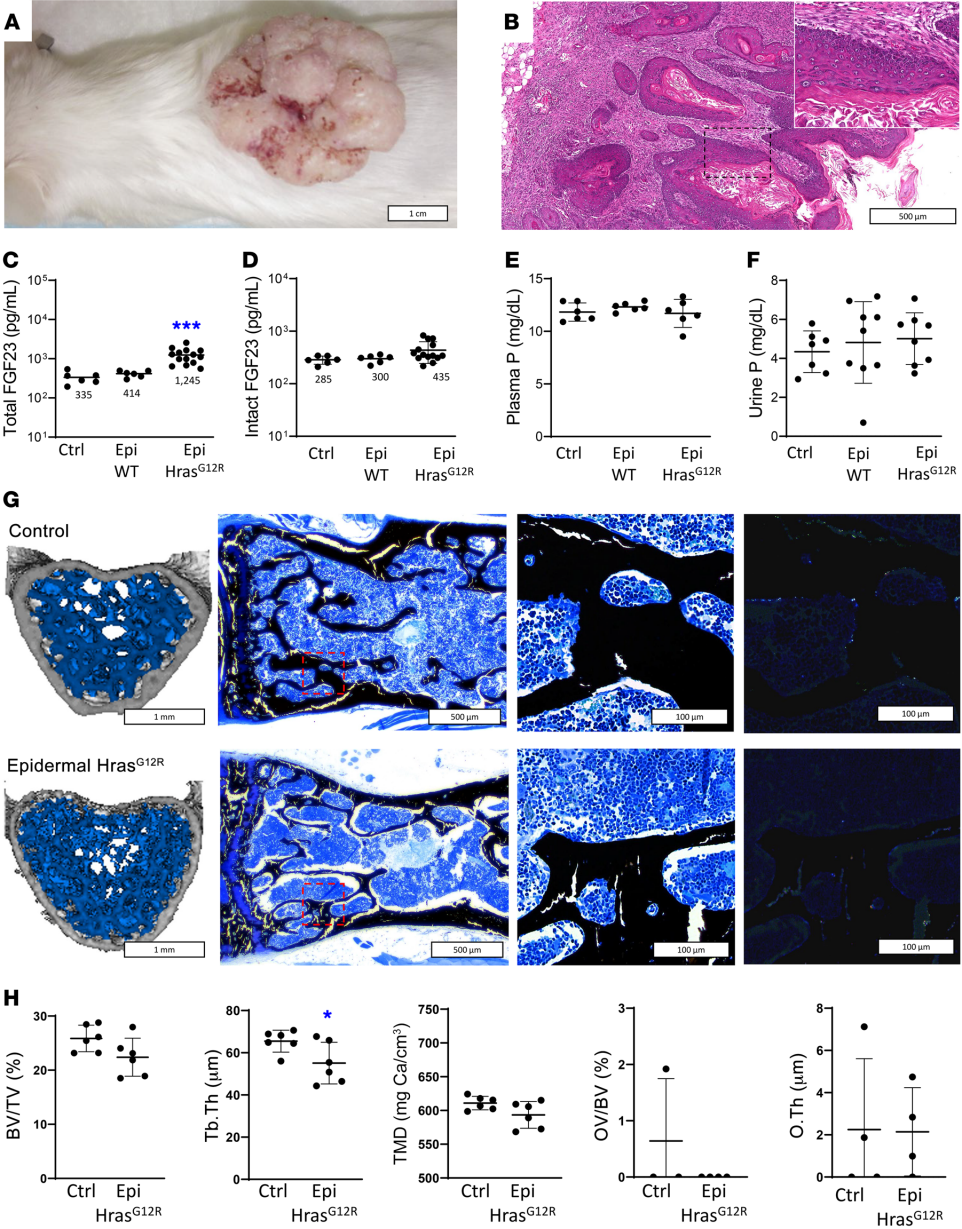
Epi Hras^G12R^ mice have a mild increase of plasma total FGF23, but no changes in intact FGF23 or phosphate levels, bone loss, or osteomalacia with respect to unaffected controls and Epi WT mice. Representative external photography (**A**) and H&E histology (**B**) of a squamous papilloma 28 days after subcutaneous transplantation with Hras^G12R^-transduced keratinocytes, showing finger-like papillary projections resulting from proliferative stratified epithelium with keratinized epithelium. The black box shows the field magnified in the top right corner. (**C**) Plasmatic levels of total (intact and C-terminal) FGF23 (*n* = 6–14). (**D**) Plasmatic levels of intact FGF23 (*n* = 6–14). (**E**) Plasma levels of phosphate (*n* = 6). (**F**) Urine levels of phosphate (*n* = 7–9). (**G**) Representative images, left to right: μCT reconstruction of L5 vertebrae, with the analyzed trabecular bone region in blue; von Kossa stain at low magnification with a red box showing the areas magnified to the left; far left shows the same areas under polarized light microscopy. (**H**) μCT bone microarchitecture parameters (*n* = 4–6). Data are shown as individual values and averages or averages with SD bars. *P < 0.05. Mann-Whitney *U* test (**C** and **D**); 1-way ANOVA with Bonferroni’s adjustment for multiple comparisons (**E** and **F**); 2-tailed Student’s *t* test (**H**).

Upon transplantation as orthografts, *Hras^G12R^*-transduced keratinocytes formed neoplastic papillary exophytic lesions at the graft site consisting of stratified keratinized epithelium with fibrovascular connective tissue cores (Figure 3, A and B). Lesion histology was similar to the EN observed in CSHS (i.e., papillomatosis, hyperkeratosis, and acanthosis), supporting it as an appropriate model for studying the effects of mutation-bearing skin lesions on phosphate metabolism in CSHS. Mice grafted with WT keratinocytes (Epi WT) developed a small area of hairless hyperplastic epidermis tissue. Although total FGF23 was 3 times higher in *Hras^G12R^* than in controls (Figure 3C), intact FGF23 levels and blood and urine phosphate were not different between Epi *Hras^G12R^*, Epi WT, and epidermal control (Epi control) mice (Figure 3, D–F). Consistently, there was no evidence of osteomalacia in Epi *Hras^G12R^* mouse L5 vertebra, although μCT analysis revealed subtle but significant trabecular thinning (Figure 3, G and H) (26).

Plasma levels of total, but not biologically active, intact FGF23 were increased in Epi *Hras^G12R^* mice, reflecting increased levels of the biologically inactive C-terminal fragment of FGF23 (Figure 3C). Increased levels of C-terminal FGF23 have been associated with systemic inflammation (27–29). Consistent with inflammation-mediated total FGF23 elevations, both Epi WT and *Hras^G12R^* mice had increased levels of the inflammation marker serum amyloid P (SAP) as well as local infiltration of CD45*^+^* cells (Figure 3 and Figure 5A).

Epi WT mice, on the other hand, did not have increased levels of total FGF23, probably due to a less pronounced inflammatory response in these mice. The grafts were smaller and not neoplastic (Epi WT tissue weighed an average of 73.5 mg, as opposed to 506.8 mg for Epi *Hras^G12R^* exophytic neoplasms). The weights of skin grafts and total or intact plasma FGF23 levels were not correlated (data not shown). Findings were similar when the mice were euthanized at 21 to 28 days after surgery.

### FGF23 production is elevated in HRAS^G12V^-expressing bone, not in Hras^G12R^-expressing skin.

**Figure 4:**
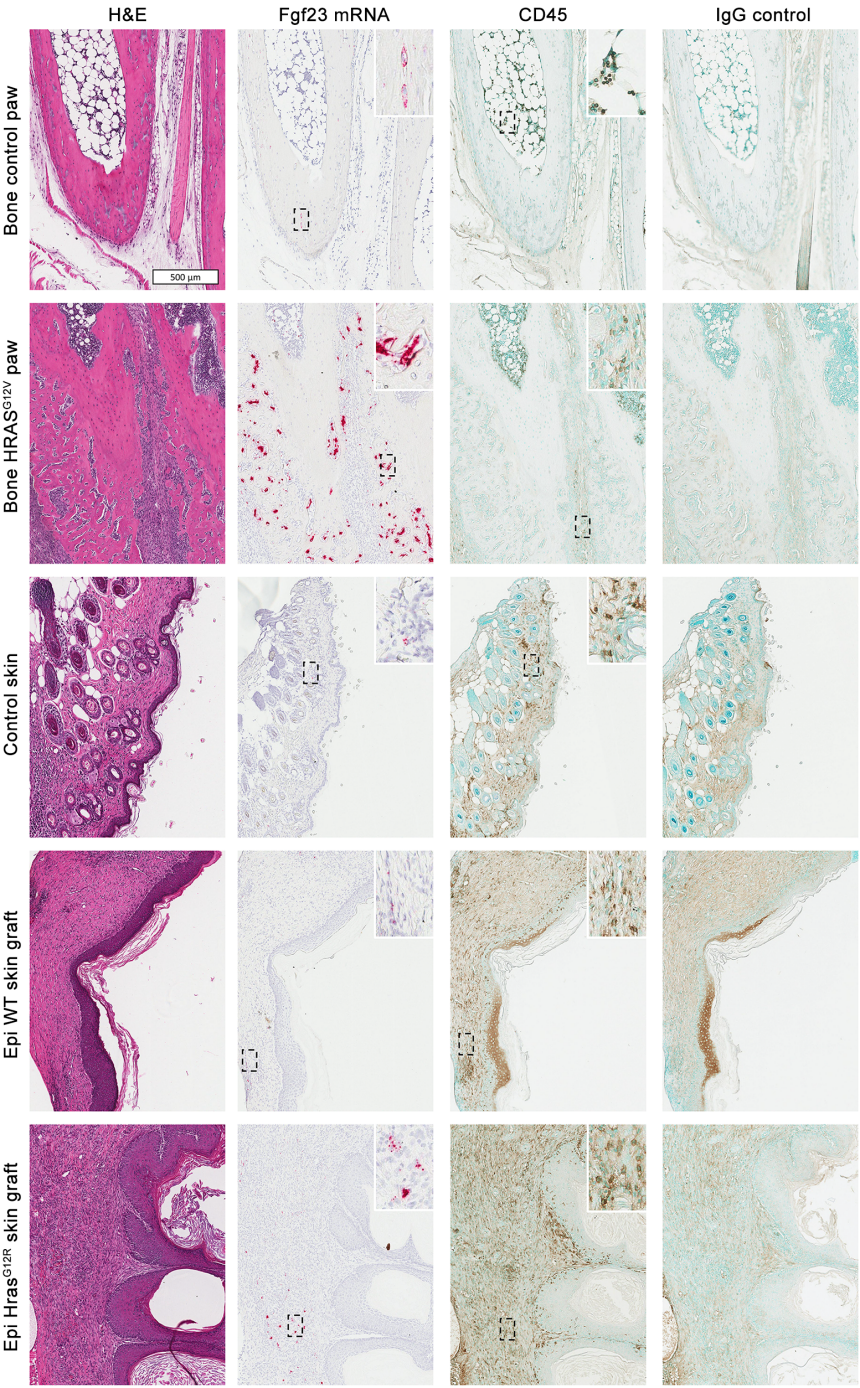
*Fgf23* expression is dramatically upregulated in bone HRAS^G12V^ dysplastic tissue and also sporadically detected in the dermal layer of skin samples. Epi Hras^G12R^– and to a lesser degree Epi WT–grafted skin, as well as the periphery of bone HRAS^G12V^ dysplastic tissue, demonstrated increased infiltration with CD45^+^ cells. From top to bottom are shown rows with representative sections of control metatarsal bone, HRAS^G12V^-expressing metatarsal bone, unaffected control skin, WT keratinocyte grated skin, and Hras^G12R^-expressing keratinocyte grafted skin. From left to right, columns show consecutive sections with H&E stain, Fgf23 mRNA, CD45 immunostaining, and negative isotype control. Positive and negative mRNA probes for Ppib and dapB, respectively, were used alongside Fgf23 probes (not shown). *n* = 5 per group. Scale bar applies to all images. Original magnification, ×400.

**Figure 5:**
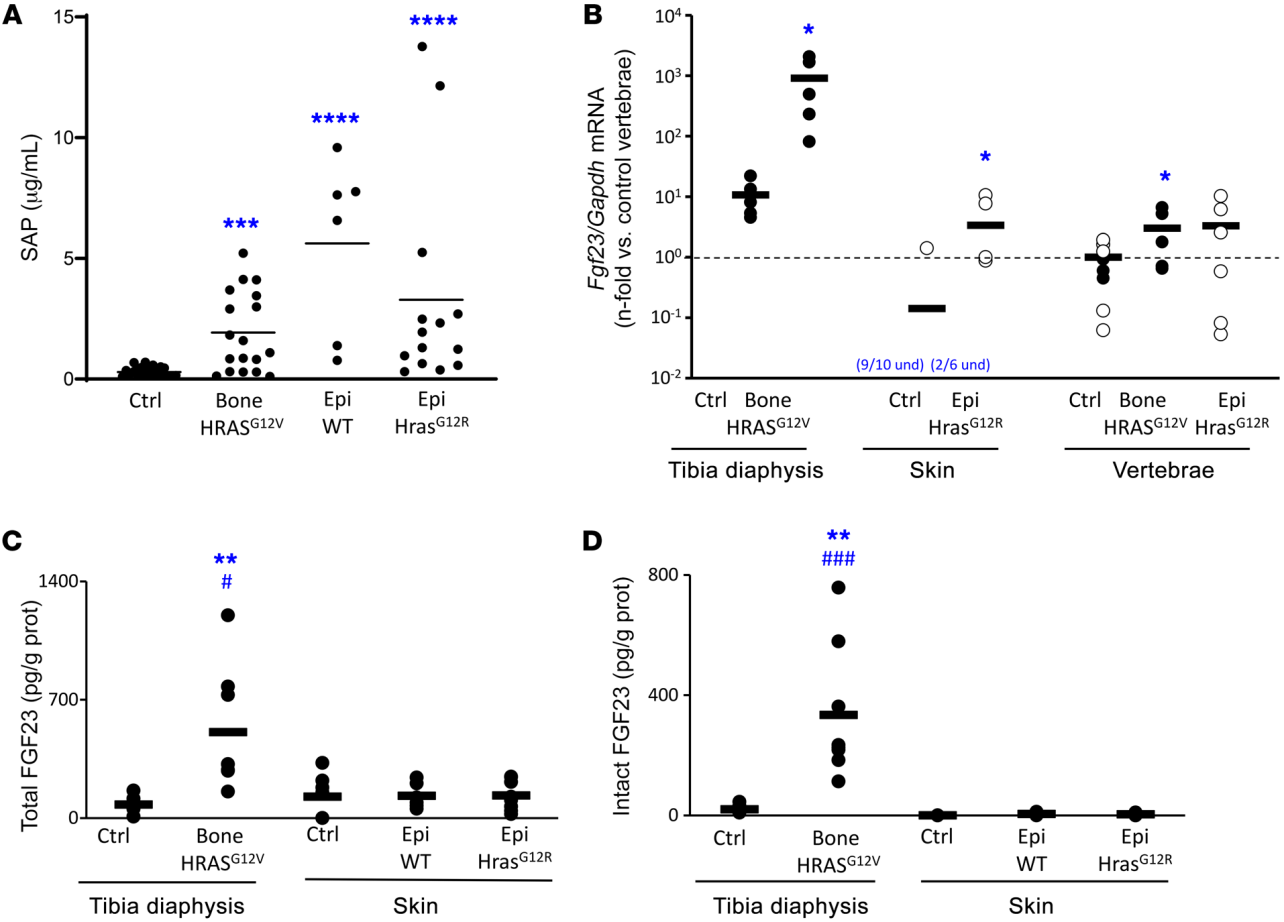
Both bone mouse models had systemic inflammation. *Fgf23* mRNA expression was markedly increased in HRAS^G12V^-expressing bone and detectable in some Epi *Hras^G12R^* grafts and skin control samples. (**A**) Plasmatic levels of SAP, an acute-phase inflammatory marker (*n* = 6–24). (**B**) qPCR showing *Fgf23* versus *Gapdh* expression in tibial cortical bone from bone HRAS^G12V^ mice and their littermate controls; affected skin from Epi *Hras^G12R^* mice and normal skin from their littermate controls; and nontransgene expressing vertebrae from both mouse models. Epidermal mouse sample individual data points are represented by white circles; bone mouse sample data points are represented by black circles; averages are represented as bars. Average *Fgf23/Gapdh* expression in vertebrae from each model’s control group was used to normalize data (*n* = 6–10). (**C**) Total FGF23 and (**D**) intact FGF23 levels in media conditioned by 48-hour incubation of cortical bone tissue from control and HRAS^G12V^-expressing tibiae as well as unaffected skin or skin grafted with WT or Hras^G12R^-expressing keratinocytes from both mouse models. FGF23 levels are normalized by total protein content in the media in pg of FGF23 per g of total protein (*n* = 6). *P < 0.05; **P < 0.01; ****P < 0.0001 versus controls. ^#^*P* < 0.05; ^###^*P* < 0.001 versus Epi Hras^G12R^. Kruskal-Wallis test with Dunn’s adjustment for multiple comparisons (**A**); 1-way ANOVA with Bonferroni’s adjustment for multiple comparisons was used for the skin samples (**C** and **D**); 2-tailed Student’s *t* test was used for all other comparisons.

*Fgf23* mRNA was stained in histological preparations of mutation-expressing and control tissues (Figure 4). As expected, mutation-expressing metatarsals in bone *HRAS^G12V^* mice showed abundant *Fgf23* transcripts. In addition, *Fgf23* expression by quantitative PCR (qPCR) in cortical bone from *HRAS^G12V^* tibiae was 2 orders of magnitude above that of control tibiae (Figure 5B); levels were also high in periosteal dysplastic tissue and bone marrow from *HRAS^G12V^* tibiae (not shown). Ex vivo cultures of affected tibiae demonstrated markedly higher levels of total and intact FGF23 secretion compared with control tibiae (507 pg of tFGF23 and 334 pg of iFGF23 per mg of protein, respectively; 16 and 6 times more than control tibiae, Figure 5, C and D).

Low levels of *Fgf23* transcripts were sporadically detected in the dermis underlining keratinocytes in Epi *Hras^G12R^* lesions and to a lesser extent in Epi WT tissue, but rarely in unaffected skin (Figure 4). Liver, muscle, and testis samples from WT mice were simultaneously stained for *Fgf23* mRNA as negative controls, and no stain was detected (data not shown). While 4 of 6 Epi *Hras^G12R^* samples and 1 of 10 control skin samples demonstrated detectable *Fgf23* mRNA by qPCR, albeit at relatively high cycle numbers (cycles 32 to 39) (Figure 5B), ex vivo culture of skin tissue showed levels of FGF23 in the media that were at or near the lower limit of detection (Figure 5, C and D). *HRAS^G12V^* bone tissue had approximately 300 times the expression of *Fgf23* mRNA in vivo and 100 times the secretion of iFGF23 ex vivo in comparison (Figure 3, B and D).

## Discussion

Elucidating the source of FGF23 excess in CSHS is important, both for understanding the pathophysiology of the disease and for improving patient management. Without sufficient evidence to support it, several authors proposed that CSHS nevi are the source of the phosphaturic factor excess and that their removal will correct renal phosphate wasting. However, a review of the literature indicates that this painful intervention is generally ineffective in controlling phosphate abnormalities (30–32) and that the improvement was only noted in the presence of confounding factors such as phosphate supplementation (7, 17). Other observations casting doubt on skin lesions as the FGF23 source are that the vast majority of patients with congenital nevi do not have hypophosphatemia and that patients with FGF23-mediated hypophosphatemia have concomitant dysplastic bone lesions (2). Furthermore, we and others have previously failed to detect FGF23 mRNA or protein in the nevi of affected patients (12, 33, 34).

In this study, using two informative mouse models, we evaluated the effects on FGF23 production, phosphate metabolism, and skeletal mineralization of gain-of-function *HRAS* mutations similar to those detected in CSHS. Bone *HRAS^G12V^* mice displayed a marked elevation of plasma-intact FGF23, phosphaturia, hypophosphatemia, and osteomalacia. None of these findings were seen in the Epi *Hras^G12R^* mice, clearly supporting the hypothesis that mutation-bearing bone lesions, and not skin nevi, are the primary source of bioactive FGF23 excess in CSHS.

The bone HRAS^G12V^ mouse was developed using the same transgenic construct validated to drive bone cell expression of *Gα_s_^R201C^* in a mouse model of FD (35). The mice conditionally expressed *HRAS*^G12V^ in the appendicular skeleton upon induction with doxycycline. The Epi *Hras^G12R^* mouse was developed as a model of squamous papilloma for studying the biochemical changes in keratinocytes relevant for tumor formation. These mutations and *HRAS^G13R^*, previously identified in CSHS patients, are functionally comparable; all have been proven to lead to impaired function of the HRAS GTPase regulatory domain, leading to constitutive signaling (1, 33). Interestingly, the skeletal lesions in bone *HRAS^G12V^* mice consistently arose from the periosteum, similar to those seen in periosteal osteosarcoma. There are too few bone specimens from patients available to make conclusions about bone histopathology in CSHS, but x-rays suggest that CSHS lesions in humans appear to arise from the periosteal/endosteal regions with spread through the marrow space, not dissimilar to what is observed in the bone HRAS^G12V^ mouse. Lesions generated by the *Hras^G12R^*-transduced keratinocyte grafts in the Epi *Hras^G12R^* mouse were histologically similar to the EN in CSHS patients, but appeared more proliferative than skin lesions in CSHS. In both cases, the differences could derive from a higher expression of *HRAS*^G12V^ and *Hras*^G12R^ transgenes in the models and/or species differences. Differences between mouse and human RAS regulation and oncogenicity are well documented (36). In patients with CSHS, the mutation event occurs during embryogenesis and is transmitted to the cellular progeny, so the nature and proportion of the surviving mutant cells in CSHS mosaic lesions are the product of a dynamic selection process that is not present in the animal models. Our attempts to induce bone expression of *HRAS^G12V^* in utero or perinatally to better mimic the natural history of CSHS resulted in in utero or perinatal death (data not shown).

Despite the differences between patients with CSHS and the mice studied here, the models were appropriate and informative. Bone *HRAS^G12V^* mice demonstrated a nearly 50-fold increase of blood-intact FGF23 levels that resulted in hypophosphatemia and hyperphosphaturia. Corresponding end-organ effects of FGF23 excess were demonstrated in the vertebrae from these mice, which represent nontransgene targeted sentinel bones chosen to study systemic skeletal effects of FGF23. Vertebrae showed rapid BMD loss after only 2 weeks of induction of *HRAS^G12V^* expression. When harvested 3 weeks later, vertebrae demonstrated osteomalacia, as evidenced by the presence of thickened osteoid seams in von Kossa staining and loss of trabecular bone volume and mineral density by μCT analysis. Taken together, these data demonstrate that mosaic-like bone expression of *HRAS^G12V^* causes FGF23-mediated hypophosphatemia and systemic osteomalacia.

Support for the feasibility of RAS signaling in regulating FGF23 is in part derived from the importance of RAS/RAF/MAPK pathway activation in FGFR1 signaling. FGFR1 is a tyrosine kinase receptor expressed in bone cells that signals primarily through the RAS/RAF/MAPK pathway, which appears to be important in FGF23 regulation. Support derives from the fact that FGF23 overproduction is a characteristic feature of osteoglophonic dysplasia, a rare form of dwarfism due to gain-of-function *FGFR1* mutations (37). In addition, *Fgfr1* up- and downregulation in various murine models corroborates the stimulatory effects of MAPK signaling in FGF23 production and expression (21, 38–40). Moreover, a significant proportion of patients with the hyper-FGF23 syndrome of tumor-induced osteomalacia (TIO) harbor an apparent driver *FN1-FGFR1* chimeric fusion gene (41, 42). The dependence of FGFR1 signaling in TIO is evidenced by the ability of the FGFR inhibitor infigratinib to markedly decrease FGF23 levels (43). All of this lends support to the hypothesis that in CSHS, *RAS*-activating mutations in bone cells lead to constitutional MAPK signaling and FGF23 overproduction.

Epi *Hras^G12R^* mice had subtle skeletal findings isolated to trabecular thinning, with no effects on overall bone and osteoid content (Figure 3F). While Epi *Hras^G12R^* mice had a 3-fold increase in circulating total FGF23, it was due to an increase in the biologically inactive catabolite C-terminal peptide. Intact FGF23, the active hormone, remained unchanged and no effects on phosphate metabolism were observed. In comparison, bone *HRAS^G12V^* mice had circulating total and intact FGF23 levels 8 and 14 times higher, respectively. This was in concordance with a local FGF23 production that was 300 times higher at the mRNA level and 100 times higher at the intact protein level than those in *Hras^G12R^* skin mice. These levels resulted in dramatic systemic bone loss in bone *HRAS^G12V^* mice and evident osteomalacia. The modest trabecular changes seen in the vertebrae of the Epi *Hras^G12R^* mice did not indicate significant bone loss and were not indicative of osteomalacia and may also be explained by chronic inflammation (26). Systemic inflammation can cause mild osteoporotic changes and lead to increased production of FGF23 that is inactivated to biologically inactive C-terminal FGF23 through furin-mediated cleavage (27–29). This explanation was supported by higher blood levels of SAP, an inflammation-associated acute phase reactant (44), and local CD45^+^ leucocyte infiltration in Epi *Hras^G12R^* mice compared with control littermates. While elevated blood SAP and local CD45^+^ cell infiltration were also detected in Epi WT mice, indicative of an inflammatory status, this is likely explained by a relatively lower inflammatory response to the grafted material in Epi WT mice, as Epi *Hras^G12R^* mouse grafts were 7 times larger.

While low levels of *Fgf23* transcripts were found sporadically in skin, the physiological significance of this remains unclear. FGF23 expression has been reported in heart, brain, small intestine, liver, kidney, spleen, thymus, and bone marrow (32, 45, 46), but it had not been reported in skin before. Based on this observation, we further assessed for the presence of *Fgf23* transcripts by qPCR in *Hras^G12V^*-transduced keratinocyte grafts in athymic mice and detected low levels of *Fgf23* expression in 2 out of 9 lesions (not shown). Thus, this represents what we believe is the first report of *Fgf23* expression in skin. It is noteworthy that RNA in situ hybridization staining revealed sporadic *Fgf23* mRNA presence in the dermis of a subset of the skin samples, but not in keratinocytes, regardless of whether they expressed *Hras^G12R^*. Instead, staining was restricted to dermal fibroblasts and higher in *Hras^G12R^* grafts. While a potential paracrine effect of mutant keratinocytes on *Fgf23* expression in neighboring dermal fibroblasts is possible, the physiological significance of this, if any, is unclear. Staining in all skin samples was far below that observed in bone *HRAS^G12V^* mice tibiae. Further, circulating intact FGF23 levels and phosphate homeostasis were unaffected in Epi *Hras^G12R^* mice. Similarly, FGF23 production ex vivo was almost undetectable, but markedly upregulated in *HRAS^G12V^*-expressing bone explants. Of note, this is the first in vitro system, to our knowledge, in which very high levels of FGF23 protein are found in the media of cells/tissues not specifically transfected with *Fgf23,* suggesting this may be a good model for further study of FGF23 transcription, translation, and posttranslational modification.

In conclusion, this study adds convincing support to the hypothesis that mosaic expression of hyperactive *HRAS* in bone, and not skin, leads to increased bioactive FGF23 secretion and, consequently, the FGF23-mediated hypophosphatemia and osteomalacia observed in CSHS. This finding not only represents an advance in our understanding of CSHS pathophysiology and the physiological regulation of FGF23, but it also provides important information for the care of patients with CSHS. Resection of skin lesions should be reconsidered in the management of the hypophosphatemia of CSHS. Medical management with either phosphate and calcitriol supplementation or the anti-FGF23 drug burosumab should be considered.

## Methods

### Mouse models.

To develop the bone *HRAS^G12V^* mice, we used a strategy similar to one we previously described in the development of a FD mouse model in which a gain-of-function *GNAS* (Gα_s_^R201C^) was expressed in a mosaic pattern in bone cells (35). In this case, substituting an inducible and appendicular skeleton targeted *HRAS^G12V^* for the *G*α*_s_^R201C^* transgene. As depicted in Figure 1, we combined 3 mouse strains: first, a tetracycline-inducible *HRAS^G12V^* mouse (TetO-HRAS strain, NCI mouse repository, 01XB4), in which cells express human *HRAS^G12V^* if in the presence of a reverse tetracycline transactivator (rtTA) and doxycycline; second, a mouse strain in which rtTA is constitutively expressed in cells coexpressing Cre recombinase and their progeny by excision of a LoxP-STOP-LoxP (LSL) sequence that prevents the expression of rtTA (ROSA26-rtTA-IRES-EGFP strain, Jackson Laboratory, 005670); third, a mouse strain that expresses Cre under the control of a *Prrx1* enhancer. *Prrx1* is expressed during embryogenesis in early limb bud mesenchyme and a subset of craniofacial mesenchyme cells (*Prx1-Cre* strain, Jackson Laboratory, 005584). As a result, and as previously shown for *G*α*_s_^R201C^* in inducible FD mice (35), these mice express *HRAS^G12V^* in cells of the skeletal lineage of the appendicular skeleton and some areas of the calvaria. At 12 weeks of age, mice were induced with doxycycline-supplemented food (100 parts per million, LabDiet, Purina). After 1 week of induction, periosteal dysplastic masses appeared in the appendicular skeleton, some of which calcified afterwards. Twenty-eight days after induction, mice were euthanized. Some mice were euthanized at an earlier time per recommendation of the facility veterinarian to avoid pain associated with swollen paws, although never before 21 days after induction. Littermate control mice lacking the tetracycline-inducible *HRAS^G12V^* transgene did not develop periosteal dysplasia and were sacrificed at day 28 after initiating the doxycycline-supplemented diet.

For the Epi *Hras^G12R^* mice, primary mouse keratinocytes and hair follicle buds were isolated from male and female newborn pups of FVB/NCr as described previously (47). On day 3 of culture, keratinocytes were infected or not with the v-ras^Ha^ retrovirus at a MOI of 1 in medium containing polybrene (4 μg/mL; Sigma-Aldrich). On day 8, cultures were trypsinized and used for grafting. Four million keratinocytes were mixed with 6 million FVB/NCr mouse primary dermal fibroblasts (cultured for 1 week) and grafted onto the dorsum skin of a 12-week-old male FVB/NCr on a prepared skin graft site. Mice were euthanized 28 days after grafting or earlier if skin lesions exceeded 2 cm in width in their longer dimension (as required by the animal protocol), but never before 21 days after transplantation. In addition, nontransformed keratinocytes were used in a similar fashion to generate *Hras^G12R^* nonexpressing grafts in 12-week-old male FVB/NCr mice (Epi WT), which resulted in a small patch of hyperplastic epidermis tissue devoid of hair follicles. Moreover, site-matched unaffected skin samples were obtained from male littermate mice and used as skin negative controls (Epi control).

### Histology and μCT.

Mice were fixed by terminal perfusion with Z-Fix (Anatech). Samples were dissected, weighed, and additionally fixed in Z-Fix at 4°C for 24 hours. Mouse hind limbs were decalcified in 250 mM EDTA in PBS pH 7.4 at 4°C. Skin and hind limbs were embedded in paraffin. Vertebrae L4–L6 were dissected and scanned using a Scanco μCT 50 at 10 μm resolution, 70 kVp, 80 μA, and 300 ms integration time. Reconstructed images were analyzed with AnalyzePro 1.0 (AnalyzeDirect). Undecalcified vertebrae were then either embedded in methylmethacrilate or Super Cryoembedding Medium (Section-Lab Co. Ltd.), frozen at –80°C, and sectioned following Kawamoto’s film method (48).

H&E and VK staining were performed using standard methods. H&E-stained samples were scanned with a NanoZoomer S60 digital slider scanner (Hamamatsu), and brightfield and polarized light images of VK-stained samples were obtained on a Leica DM2500 microscope. Osteoid histomorphometry was evaluated using the Bioquant Osteo Software Suite, version 17.2.6.

*Fgf23* mRNA was detected by in situ hybridization using the RNAscope System (Advanced Cell Diagnostics [ACD]). Briefly, paraffin sections were hydrated, incubated with RNAscope Hydrogen Peroxide Solution (ACD, 322335), and retrieved using the ACD Custom Pretreatment Reagent (ACD, 300040) for 45 minutes at 40°C. Serial sections were incubated with probes against *Fgf23* (ACD, 444541), *Ppib* (positive control probe, ACD, 313911), or *dapB* (negative control probe, ACD, 310042) for 2 hours at 40°C. The remaining hybridization steps were performed using the RNAscope 2.5 HD Detection Reagents-RED Kit according to the manufacturer’s instructions, except for AMP5, which was increased to 45 minutes to improve staining intensity. Sections were counterstained with 50% Gil’s Hematoxylin I (Sigma-Aldrich, GHS132) and mounted with EcoMount Mounting Medium (Biocare Medical, EM897L).

For CD45 immunohistochemistry, paraffin sections were hydrated and endogenous peroxidase was blocked with 3% H_2_O_2_ in methanol for 10 minutes at room temperature (RT). Antigen retrieval was performed with Uni-Trieve (Innovex Biosciences, NB325) for 30 minutes at 55°C. Sections were blocked using rabbit serum (Vector, PK-4004) with 2.5% bovine serum albumin for 30 minutes at RT and then incubated with rat anti-CD45 (1:1000, BD Biosciences, catalog 550539) or rat IgG isotype control (1:1000, Vector, I-4000-1) for 2 hours at 4°C. Next, sections were incubated with biotinylated rabbit anti-rat IgG for 45 minutes at RT and VECTASTAIN Elite ABC Reagent (Vector, PK-4004) for 30 minutes at RT and stained using DAB-EASY tablets (Acros Organics, Thermo Fisher Scientific, AC328005000). Samples were counterstained with Methyl Green (Vector, H3402-500) and mounted using EcoMount.

### Dual-energy x-ray absorptiometry, x-rays.

Dual-energy x-ray absorptiometry (DEXA) analysis of bone *HRAS^G12V^* and control mice as well as x-ray images were obtained using a Faxitron Ultrafocus. Total body DEXA scans were performed at automatically selected exposures that ranged between 41.0 and 43.0 Kv, and L4 and L5 vertebrae were analyzed with Bioptics Vision DEXA 2.3. X-rays were obtained at 40 Kv for 2.2 seconds (Faxitron Bioptics LLC-Vision NDT, version 2.3.1).

### qPCR.

Tibia were dissected from bone *HRAS^G12V^* and control mice, and samples of mid-diaphyseal cortical bone, bone marrow, and hyperplastic periosteal tissue (in bone *HRAS^G12V^* mice) were obtained and snap-frozen in dry ice. Skin samples from Epi *Hras^G12R^* keratinocyte grafts and control littermates were obtained and snap-frozen in dry ice. Whole L5 vertebrae were dissected and snap-frozen in dry ice. RNA was extracted from all samples using the phenol-chloroform method, and cDNA was generated using the iScript cDNA Synthesis Kit (Bio-Rad). qPCR of *Fgf23* and *Gapdh* was performed using TaqMan probes (mm00445621_m1, #m99999915_g1, respectively; Thermo Fisher) on a QuantStudio 3 qPCR System (Thermo Fisher). Data are expressed as *ΔC_t_* *Fgf23/Gapdh* and normalized with respect to each model’s vertebrae tissue (=1 arbitrary units).

### FGF23 secretion ex vivo.

Pieces of approximately 100 mg of epidermal tissue were dissected from Epi *Hras^G12R^* grafts and Epi WT grafts 28 days after transplantation and control skin from unaffected littermates. Similarly, tibias weighing 100 to 120 mg were extracted from bone *HRAS^G12V^* and control mice 28 days after induction with doxycycline. All tissues were cut in pieces of approximately 1 to 8 mm^3^ and incubated in 12-well plates with 1 mL of α-MEM medium (Thermo Fisher Scientific) with 0.1% of Normicin (Invivogen, ant-nr-1) in a culture incubator. Twenty-four hours later, medium was replaced by fresh medium, which was collected 48 hours later and centrifuged at 15,000*g* for 5 minutes to remove solids. Protein was measured in samples diluted 1:10 in PBS using the Coomassie method (Coomassie Plus, Thermo Fisher Scientific, 23236). Protein standards were BSA solutions of 25 to 2000 μg/mL in 1:10 media:PBS (Thermo Fisher Scientific, 23209). FGF23 levels were normalized by protein content in the media.

### Urine, plasma, and media measurements.

Urine and blood were collected, and heparinized plasma was obtained. Phosphate was measured either by IDEXX BioAnalytics or by the Department of Laboratory Medicine at the NIH Clinical Center.

Total FGF23 (C-terminal fragments plus intact) was measured by ELISA (Quidel, 60-6300). Intact FGF23 was measured by ELISA (Kainos, CY-4000). For ex vivo media measurements, appropriate media blanks were used. SAP was measured using ELISA kit LSBio LS-6414 (LIfeSpan Biosciences).

### Statistics.

Data are represented as individual data points and mean ± SEM for all values. Results were tested for normality using the Shapiro-Wilk test. One-way ANOVA with Bonferroni’s adjustment for multiple comparisons was used for Figure 3, E and F, and Figure 5, C and D (only for the skin samples), and Kruskal-Wallis test with Dunn’s adjustment for multiple comparisons was used for Figure 3, C and D, and Figure 5A. Comparisons in Figure 2, D and E, were evaluated with the Mann-Whitney *U* test. The remaining comparisons were evaluated using 2-tailed, unpaired Student’s *t* test. Analysis was performed using GraphPad Prism, version 8.0.2, software (GraphPad Software). *P* < 0.05 was considered significant.

### Study approval.

Animal studies were conducted under protocols approved by the Animal Care and Use Committees of NIDCR (Animal Study Procedure 19-897) and the NCI (Animal Study Procedure LCCTP-053).

## Author contributions

DO developed the bone HRAS^G12V^ mouse model. DO and ZM performed experiments, interpreted data, and contributed to writing the paper. CC designed and performed experiments. AS performed experiments. RG designed and performed experiments. SHY interpreted data. LFDC designed and performed experiments, interpreted data, and contributed to writing the paper. MTC conceived the project, designed experiments, interpreted data, and contributed to writing the paper. DO appears before ZM in the author list because she developed the bone HRAS^G12V^ mouse model and ZM carried on with the project.

## Version 1 03/21/2023

In-Press Preview

## Version 2 05/01/2023

Electronic publication
